# Electrocortical Reactivity to Emotional Faces in Youth of Depressed Mothers: The Moderating Role of Emotion Regulation Styles

**DOI:** 10.1007/s10802-025-01417-8

**Published:** 2026-01-13

**Authors:** Caley Lane, Eda Naz Dinc, Chuck Kingston, Cope Feurer, Katie L. Burkhouse

**Affiliations:** 1 The Research Institute, Nationwide Children’s Hospital, Columbus, OH USA; 2 Department of Psychology, 259 Moore Building, Pennsylvania State University, University Park, State College, Pennsylvania, PA 16801 USA; 3 Department of Psychiatry and Behavioral Health, The Ohio State University, Columbus, OH USA; 4 Department of Psychiatry, University of North Carolina Chapel Hill, Chapel Hill, NC USA

**Keywords:** Late positive potential, Emotion regulation, Rumination, Maternal depression, EEG

## Abstract

Children of depressed mothers are at significantly high risk (HR) for developing major depressive disorder (MDD) compared to their low risk (LR) counterparts. Evidence shows that HR youth exhibit a reduced late positive potential (LPP), an electroencephalogram (EEG) marker of emotional reactivity, in response to social-emotional stimuli. However, it remains unknown how emotion regulation (ER) styles may impact LPP responses in HR and LR offspring. The current study sought to examine the interplay of ER strategies (i.e., rumination, cognitive reappraisal, and suppression) and maternal history of MDD in association with LPP responses to emotional stimuli among youth. Participants included 112 mother-child dyads (child age range 9–16 years) participating in a larger study on the intergenerational transmission of depression. Mothers either had a history of MDD (*n* = 67) or no history of psychopathology (*n* = 45). Youth completed an emotional face matching task while EEG was recorded to measure the LPP. Participants completed self-report measures of ER strategies and depressive symptoms. Results revealed an interactive effect of maternal MDD history and youth rumination on child’s LPP response. Specifically, a more blunted LPP response to emotional faces and shapes was observed among HR youth who engaged in more frequent rumination. Results revealed no main or interactive effects of youth’s use of cognitive reappraisal or suppression strategies in shaping youth’s LPP response. Findings suggest that certain characteristics among HR offspring (e.g., reporting more frequent use of rumination) may place them at higher risk for exhibiting attenuated emotional reactivity at the neural level.

## Introduction

Offspring of depressed mothers are at significantly higher risk for developing major depressive disorder (MDD), compared to offspring of never depressed mothers (Goodman, 2007; Hammen, 2009; Silver et al., 2020). Moreover, maternal MDD is associated with an earlier age of onset, a more chronic course, and a higher risk of recurrence of MDD in offspring (Goodman, 2007; Hammen, 2009; Weissman et al., 2016). Maternal MDD has also been linked to a plethora of both internalizing and externalizing symptoms in offspring, impacting multiple domains of functioning (Goodman & Tully, 2006). Given the maladaptive outcomes that have been observed in these high risk (HR) youth of depressed mothers, it is critical to identify potential risk factors involved in the intergenerational transmission of depression to develop targeted prevention efforts for this vulnerable population. In particular, studying risk factors during late childhood through middle adolescence among offspring of depressed mothers is especially critical given that symptoms and diagnoses of depression increase during this key developmental period (Kessler et al., 2001).

Alterations in emotion processing represent one risk factor that may place offspring of depressed mothers at heightened risk for experiencing depression across development (Goodman, 2007; Goodman & Gotlib, 1999). Emotion processing encompasses attentional, reactivity, and regulation components (Beauchaine, 2015; Gross & Jazaieri, 2014). Numerous studies show that alterations in emotion processing play a crucial role in the development and maintenance of a wide range of psychopathologies, and are also known to prospectively predict psychopathology in HR offspring (Aldao & Dixon-Gordon, 2014; Sloan et al., 2017; Ashman et al., 2008; Dawson et al., 2003; Feng et al., 2012; Lopez-Duran et al., 2012). One way of studying emotion processing in HR offspring is by using event-related potentials (ERP) derived from electroencephalogram (EEG) (Burkhouse & Kujawa, 2023). In particular, the late positive potential (LPP) is one ERP that specifically measures sustained attention and reactivity to social-emotional stimuli (Sabatinelli et al., 2007). In children and adolescents, the LPP can be observed around 500 milliseconds following stimulus onset at parietal-occipital sites and is typically more enhanced for emotional stimuli relative to neutral stimuli (Hajcak & Dennis, 2009; Kujawa et al., 2015). The LPP can also be divided into early (e.g., 500-1,000 ms) and later (e.g., > 1000 ms) components (e.g., Hajcak & Dennis, 2009), with the early LPP thought to reflect heightened attention to motivationally salient stimuli and the later time-window indexing deeper affective processing (MacNamara et al., 2009). Emerging evidence in the LPP literature suggests that HR youth are characterized by a blunted LPP in response to social-emotional stimuli. For example, studies have shown that HR children display a blunted LPP to emotional faces compared to children of non-depressed mothers (Kujawa et al., 2012; Seidman et al., 2020). Similarly, adolescents of depressed parents, versus non-depressed parents, exhibit blunted LPP responses to both emotional and neutral images (Nelson et al., 2015). However, in one study, HR youth were characterized by an enhanced LPP response to negative self-referential words (Speed et al., 2016), suggesting that this pattern might reverse in youth of depressed mothers when processing negative information that is personally salient.

There is also emerging evidence in the literature that not all offspring of depressed mothers exhibit alterations in emotion processing at the electrocortical level, suggesting the presence of factors that may exacerbate or mitigate risk patterns. For instance, there is evidence that altered LPP responses are most pronounced among offspring of depressed mothers exhibiting higher clinical symptoms or exposed to negative environmental experiences (e.g., childhood maltreatment, poor parenting) (for a review, see Burkhouse & Kujawa, 2023). Individual differences in self-reported everyday use of emotion regulation (ER) strategies, such as rumination, cognitive reappraisal, and suppression, may also impact electrophysiological patterns of emotion processing in youth. Rumination, defined by habitual fixation on one’s symptoms, causes, and consequences of past emotional distress (Nolen-Hoeksema, 1991), is one specific risk factor for the development of depression and anxiety symptoms and disorders in youth (Burwell & Shirk, 2007; Nolen-Hoeksema et al., 2008; Wilkinson et al., 2013; McLaughlin & Nolen-Hoeksema, 2011) and may impact the LPP response. For instance, in two separate studies, researchers have found that higher trait and state-induced rumination were associated with a more enhanced LPP to loss stimuli (i.e., losing money) in a reward processing task (Webb et al., 2017) and negative scenes (Lewis et al., 2015), respectively, suggesting that those with a propensity to ruminate also show altered LPP responses. However, in a separate study, researchers found that anxious youth who engage in more frequent rumination in daily life exhibited an attenuated LPP response to unpleasant images (Bylsma et al., 2022), suggesting that higher rumination may reflect greater neural disengagement from emotional stimuli. Although prior studies have shown that HR, versus LR, offspring report using rumination on a more frequent basis (Gibb et al., 2012), no studies have examined how habitual rumination may impact the LPP response in this population.

Suppression refers to the inhibition of outward emotional expressions, as well as the internal suppression of emotions and thoughts (Gross & Thompson, 2007). Similar to rumination, habitual suppression has also been associated with depression and anxiety symptoms in youth (Schäfer et al., 2017). Two studies have evaluated the effect of expressive suppression on the LPP in small college student samples. In these studies, participants were instructed to engage in expressive suppression in response to high and low intensity pleasant stimuli (Li et al., 2020) and sad scenes (Yan et al., 2022) and found that expressive suppression attenuated the LPP. However, it is not clear how habitual rumination and suppression may affect the LPP in youth at high and low risk for MDD.

In contrast to rumination and suppression, habitual cognitive reappraisal has generally been found to be an adaptive ER style and involves changing the interpretation of an affective stimulus to modify an emotional response (Aldao et al., 2010). A large body of research shows that higher cognitive reappraisal in youth is associated with lower depressive symptoms (Schäfer et al., 2017; Shapero et al., 2019). Furthermore, looking at HR offspring specifically, these youth have been shown to report using reappraisal on a less frequent basis, relative to LR peers (Loechner et al., 2020). Moreover, Kudinova and colleagues (2018) found that among children with a parental history of MDD, those who reported using cognitive reappraisal exhibited lower rates of lifetime depressive disorders and higher levels of positive affect. Electrocortical studies with adult participants have shown that the habitual use of cognitive reappraisal is associated with a reduced LPP both when passively viewing threatening images (Harrison & Chassy, 2019) and when asked to engage in positive reappraisal while viewing negative, high-arousal pictures in the laboratory (Moser et al., 2014). It is important to note that in these studies (Harrison & Chassy, 2019; Moser et al., 2014), the LPP reductions did not take effect until after 1,000 milliseconds, the deeper stage of processing, which can be interpreted as successful emotion regulation. On the other hand, other work has shown that more frequent use of reappraisal was associated with increased early LPP during passive viewing of aversive pictures, possibly reflecting a heightened emotion regulation preparedness (Zehtner et al., 2023). Additionally, other studies found no association between reappraisal frequency and the LPP in response to negative images in youth with anxiety (Bylsma et al., 2022) or in adults with and without social anxiety disorder (Kinney et al., 2019). Thus, these studies demonstrate mixed findings on habitual cognitive reappraisal and the LPP, with findings showing an association between reappraisal and an increased early LPP (Zehtner et al., 2023) and reduced late LPP (Harrison & Chassy, 2019; Moser et al., 2014), while other work found no association (Bylsma et al., 2022; Kinney et al., 2019). Moreover, it remains unknown how individual differences in self-reported, habitual ER styles may impact the association between maternal history of depression and LPP response in offspring.

Taken together, the current study sought to examine whether self-reported, habitual rumination, suppression, and cognitive reappraisal interacted with maternal history of MDD to predict LPP response to emotional stimuli in offspring. The present study focused on offspring aged 9 to 16 years, as this developmental period represents a window in which emotion regulation abilities develop substantially and vulnerability to depression and other internalizing disorders increase, particularly among high risk youth of depressed mothers (Young et al., 2019). Furthermore, neural systems involved in affective processing also develop throughout adolescence (see Ladouceur, 2012 for a review), and targeting this age range of 9 to 16 years can allow us to identify early-emerging neural markers that may signal vulnerability and inform early intervention efforts. Consistent with previous literature, we expected that offspring of depressed mothers would be characterized by an attenuated LPP response to emotional stimuli (i.e., faces), compared to offspring of non-depressed mothers. However, we further predicted that this effect would be amplified (more attenuated LPP) in offspring of depressed mothers reporting more maladaptive ER strategies (i.e., suppression, rumination), while the effect would be reduced in HR youth reporting more frequent use of cognitive reappraisal.

## Method and Materials

### Participants and Procedures

Participants included 112 youth ages 9–16 years (*M*_age_ = 12.66 years, *SD* = 2.11, 78% female). 67 youth were at high risk for MDD based on biological maternal history of MDD and 45 youth were at low risk for MDD. The mothers (*M*_age_ = 42.75 years, *SD* = 6.38) of the participating youth lived in the same household as the child. Youth identified as White (56%), Black (15%), Asian (12%), other or unknown race (12%), and biracial (5%). Approximately 32% of youth identified as Hispanic/Latine. Youth were enrolled across two studies on the intergenerational transmission of maternal depression. Recruitment strategies, study procedures, and research personnel were identical across studies. Youth were recruited in a large Midwestern metropolitan city using flyers, community events, and internet postings (e.g., Facebook).

Psychiatric diagnoses for mothers and adolescents were obtained using the Structured Clinical Interview for DSM-5 (SCID; First et al., 2015) and Schedule for Affective Disorders and Schizophrenia for School-Age Children (K-SADS-PL; Kaufman et al., 2016), respectively. Of the 112 youth, 67 had mothers with a history of MDD (9 with current MDD), and 45 had mothers with no DSM-5 psychiatric illness histories. Study exclusions for youth included having substance/alcohol dependence within the past 6 months or histories of bipolar disorder, schizophrenia, intellectual disability, serious medical conditions, and pervasive developmental disorders. Youth with a current depressive episode and active suicidal ideation were also excluded, as the larger studies in which the data were collected from aimed to understand vulnerability markers for youth depression with a longitudinal design. Of the youth in the study, the following mood disorders were endorsed: past MDD (HR, *n* = 6), past persistent depressive disorder (HR, *n* = 1), and past disruptive mood dysregulation disorder (HR, *n* = 1; LR, *n* = 1). In addition, the following anxiety disorders were endorsed: past panic disorder (HR, *n* = 2), past generalized anxiety disorder (HR, *n* = 1), current generalized anxiety disorder (HR, *n* = 13; LR, *n* = 4), current social anxiety disorder (HR, *n* = 4; LR, *n* = 2), current specific phobia (HR, *n* = 2), and past post-traumatic stress disorder (HR, *n* = 1). Finally, the following other disorders were endorsed: current attention-deficit hyperactivity disorder (ADHD) inattentive type (HR, *n* = 7; LR, *n* = 4), current ADHD hyperactivity type (HR, *n* = 1), current ADHD combined type (HR, *n* = 2), and past oppositional defiant disorder (HR, *n* = 2).

The following other disorders were endorsed by the mothers with MDD in the study: alcohol use disorder (lifetime *n* = 11), substance use disorder (lifetime *n* = 5), anorexia nervosa (lifetime *n* = 3), bulimia nervosa (lifetime *n* = 2, current *n* = 1), panic disorder (lifetime *n* = 7, current *n* = 3), agoraphobia (lifetime *n* = 3, current *n* = 3), social anxiety disorder (lifetime *n* = 15, current *n* = 14), generalized anxiety disorder (lifetime *n* = 16, current *n* = 6), specific phobia (lifetime *n* = 4, current *n* = 4), and post-traumatic stress disorder (lifetime *n* = 5, current *n* = 4).

### Measures

#### Emotion Regulation Styles

Youth completed the Ruminative Response Scale (RRS; Treynor et al., 2003) to measure rumination. The RRS is a 22-item self-report measure with higher scores indicating higher levels of rumination. The RRS demonstrated excellent internal consistency in the current study (α = 0.93). Youth also completed the Emotion Regulation Questionnaire (ERQ; Gross & John, 2003). The ERQ is a 10-item self-report measure with one subscale assessing cognitive reappraisal and the other assessing suppression. The subscales demonstrated adequate internal consistency estimates (cognitive reappraisal α = 0.83, suppression α = 0.67).

#### Depressive Symptoms

Children’s symptoms of depression were assessed using the Children’s Depression Rating Scale-Revised (CDRS-R; Poznanski & Mokros, 1996). The CDRS-R is a 17-item interviewer-administered measure and has demonstrated excellent reliability and validity in previous research (e.g., Mayes et al., 2010; Poznanski & Mokros, 1996). The summary score (interviewer-derived consensus among parent and child scores) was used in analyses and demonstrated adequate internal consistency (α = 0.67). Given that the CDRS-R was administered to some families virtually during the COVID-19 pandemic, observational items 15–17 were omitted from the total score.

#### Emotional Face-Matching Task

Participants completed an emotional face-matching task (Kujawa et al., 2015; MacNamara et al., 2013). The task involved the presentation of three images in a triangular arrangement for 3000 ms and participants were instructed to select which of the two images at the bottom of the screen matched the image centered at the top of the screen. The task included both face matching and shape matching trials. On face matching trials, one emotional face was presented at the top of the screen (angry, fearful, happy, or sad face), and other faces with a similar expression or a neutral expression were presented at the bottom of the screen. Participants selected which emotional face at the bottom of the screen matched the expression of the face at the top. Shape matching trials were included to examine ERPs in a non-emotional condition and required participants to match geometric shapes. The task included 12 trials for angry, fearful, happy, and sad faces, and 32 trials for shapes. The intertrial interval varied between 1000 and 3000 ms during which a white fixation cross was presented on a black background. Participants performed three practice trials prior to beginning the experiment.

#### EEG Data Acquisition and Preprocessing

Continuous EEG was recorded using a 34-channel cap (32 channel setup based on 10/20 system with the addition of FCz and Iz) and the BioSemi system (BioSemi, Amsterdam, Netherlands). Electrodes were placed on the left and right mastoids, and the electrooculogram (EOG) was recorded from four facial electrodes placed approximately 1 cm above and below the right eye and beyond the outer edge of each eye. The data were digitized at 24-bit resolution with a Least Significant Bit (LSB) value of 31.25 nV and a sampling rate of 1024 Hz, using a low-pass fifth order sinc filter with − 3dB cutoff point at 208 Hz. As per BioSemi’s design, the voltage from each active electrode was referenced online with respect to a common mode sense active electrode producing a monopolar (non-differential) channel.

**Fig. 1:**
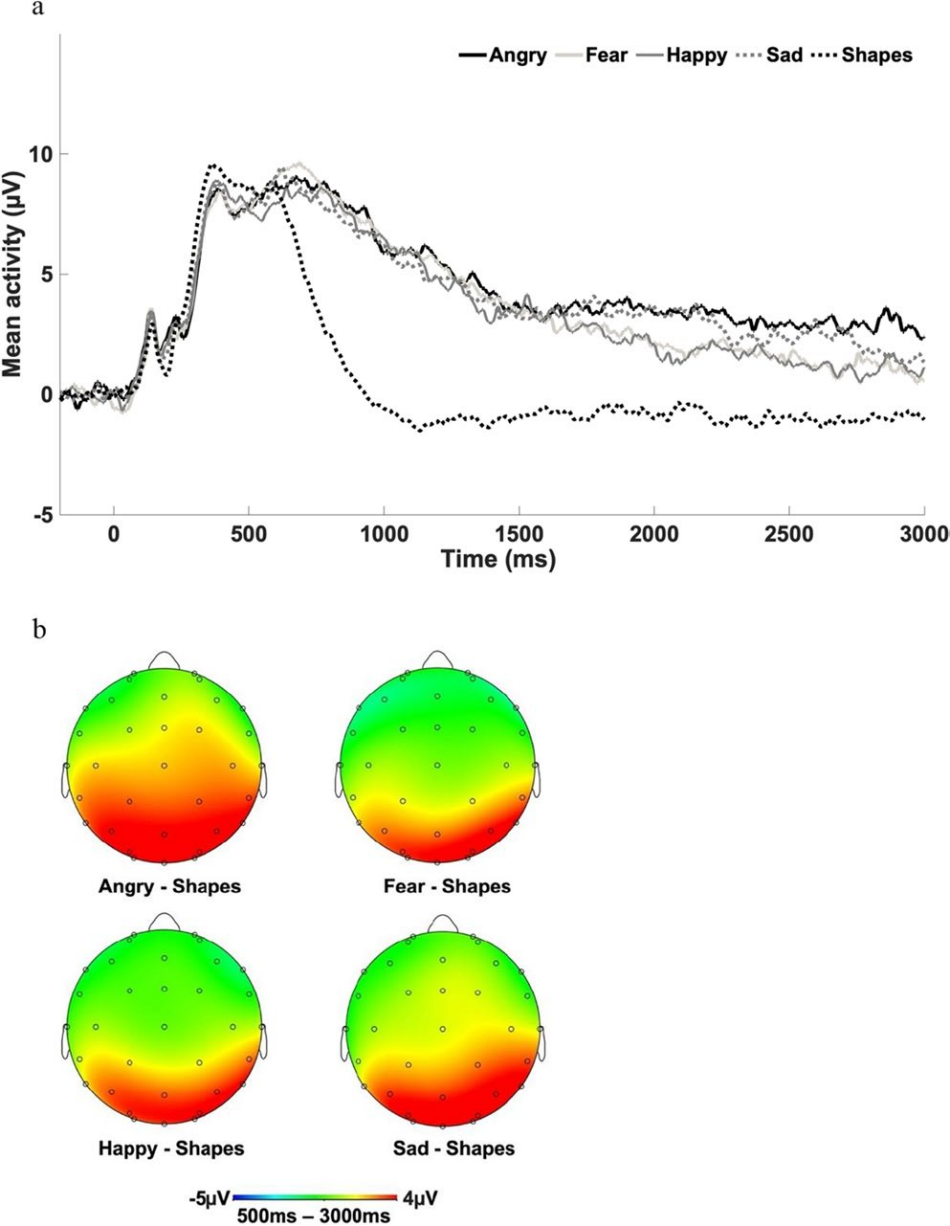
*Waveform (1a) and scalp topographies (1b) depicting the late positive potential (LPP) in response to emotional faces across all participants. The LPP in the waveform is pooled from the following electrodes: O1*,* O2*,* Oz*,* PO3*,* PO4*,* P3*,* P4*,* and Pz*

Data were processed offline using Brain Vision Analyzer software (Brain Products, Gilching, Germany). Data were converted to a linked mastoid reference, filtered with high-pass and low-pass filters of 0.01 and 30 Hz, respectively. Data were segmented beginning 200 ms before stimulus onset and continuing for the 3000 ms stimulus duration. Eyeblinks were corrected using the method by Gratton et al. (1983), and semi-automated artifact rejection procedures removed artifacts with the following criteria: voltage step of more than 50 µV between sample points, a voltage difference of 300 µV within a trial, and a maximum voltage difference of less than 0.5 µV within 100 ms intervals. Additional artifacts were removed using visual inspection. Participants were required to have a minimum of 8 artifact-free trials in at least four electrodes used to create the LPP for each condition to be included in analyses (Moran et al., 2013). Trials were baseline corrected using the 200 ms prior to stimulus onset and averaged across each condition (shapes, angry, fearful, happy, sad). The LPP was maximal across parietal and occipital sites, consistent with previous LPP research with youth (Kujawa et al., 2015), and scored at a pooling of the following electrodes: O1, O2, Oz, PO3, PO4, P3, P4, and Pz (see Fig. 1). Consistent with previous studies, the LPP was divided into two separate windows (early: 500–1000 ms, late: 1000–3000 ms).

### Analytic Plan

To examine the interplay of ER styles and group on youth’s LPP, separate Condition (fearful, happy, sad, neutral, shapes) × LPP Time Window (early: 500–1000 ms, late: 1000–3000 ms) mixed ANOVAs were conducted. In these ANOVAs, group was entered as a between-subject variable and ER [i.e., total rumination (RRS) score, suppression (ERQ-suppression) score, or reappraisal (ERQ-reappraisal) score] was entered as covariate of interest. Child age and gender were also entered as covariates. Condition × Time Window × Group × ER interactions were tested with LPP serving as the dependent variable. Post-hoc analyses were conducted to test whether significant effects remained significant after adjusting for the influence of child depressive symptoms.

## Results

As shown in Table 1, groups reported similar levels of reappraisal, rumination, and suppression, but youth of depressed, relative to non-depressed, mothers exhibited higher depressive symptoms. A correlation table of key study variables is provided in Table 2.

**Table 1:** Differences in key study demographics and variables by group

	Low-Risk (*n*.= 45)	High-Risk (*n* = 67)	
	M (SD)	M (SD)	*t*
Age	13.11 (2.16)	12.36 (2.04)	−1.87
RRS	34.89 (10.85)	35.12 (10.05)	0.12
ERQ-Reappraisal	28.80 (8.01)	28.13 (8.52)	−0.41
ERQ-Suppression	14.29 (5.18)	14.13 (5.33)	−0.15
CDRS-R	16.75 (3.58)	18.12 (4.40)	1.72
	*Median*	*Median*	*t*
Family Income	90,000	75,000	−1.82
	*N (%)*	*N (%)*	*χ*^*2*^
Female	38 (84%)	49 (73%)	1.99
Race			3.41
White	27 (60%)	36 (53%)	
Black	5 (11%)	12 (18%)	
Asian American	7 (16%)	6 (9%)	
Biracial	1 (2%)	5 (7%)	
Other/Unknown	5 (11%)	8 (12%)	
Hispanic/Latine	12 (27%)	24 (35%)	1.03

*RRS* = Ruminative Response Scale, *ERQ* = Emotion Regulation Questionnaire, *CDRS-R* = Children’s Depression Rating Scale-Revised

**Table 2:** Correlations among key study variables

	1.	2.	3.	4.	5.	6.	7.
1. Child Age	-						
2. Maternal MDD	− 0.17	-					
3. Child Sex (% female)	0.29**	− 0.13	-				
4. ERQ-Reappraisal	0.04	− 0.04	0.15	-			
5. ERQ-Suppression	0.07	− 0.01	− 0.22*	0.01	-		
6. RRS Total	0.28**	0.01	0.06	− 0.12	0.40**	-	
7. CDRS-R	0.11	0.16	0.02	− 0.23*	0.09	0.45**	-
8. Average LPP	− 0.34**	0.06	− 0.16	− 0.03	0.09	− 0.13	− 0.10

*MDD* = Major Depressive Disorder, *ERQ* = Emotion Regulation Questionnaire, *RRS* = Ruminative Response Scale, *CDRS-R* = Children’s Depression Rating Scale – Revised. Average LPP = Late Positive Potential Response across all Conditions and Times. ^*^p <.05; ^**^p <.01; ^***^p <.001

To evaluate task effects, we used a generalized linear model repeated measures ANOVA with LPP amplitudes for each condition type (angry, fear, happy, sad, shapes) and time (early: 500 to 1000ms, late: 1000 to 3000ms) serving as the within-subject variables. Results revealed a main effect of Condition, *F* (4, 108) = 18.21, *p* <.001, *η*_*p*_^2^ = 0.14), and Time, *F* (1, 111 = 264.67, *p* <.001, *η*_*p*_^2^ = 0.71). As expected, the LPP response across conditions was higher during the early (*M* = 7.25, *SE* = 0.39), versus the late (*M* = 2.33, *SE* = 0.33), LPP window. To investigate the main effect of Condition, we conducted Bonferroni pairwise post-hoc comparisons. Results indicated that the LPP in response to shapes was significantly smaller than those observed for the LPP to angry (mean difference = − 4.68; CI = − 6.30, − 3.06, *p* <.001), fearful (mean difference = − 4.18; CI = − 5.82, − 2.54, *p* <.001), happy (mean difference = − 3.81; CI = − 5.49, − 2.13, *p* <.001), and sad (mean difference = − 4.06; CI = − 5.66, − 2.45, *p* <.001) faces. No other post-hoc comparisons reached significance (*p*’s = 1.00). These findings are displayed in the waveform in Fig. 1 along with the scalp topographies for each condition. The Condition × Time interaction was not significant, *F* (4, 108) = 1.79, *p* =.13, *η*_*p*_^2^ = 0.02).

**Fig. 2:**
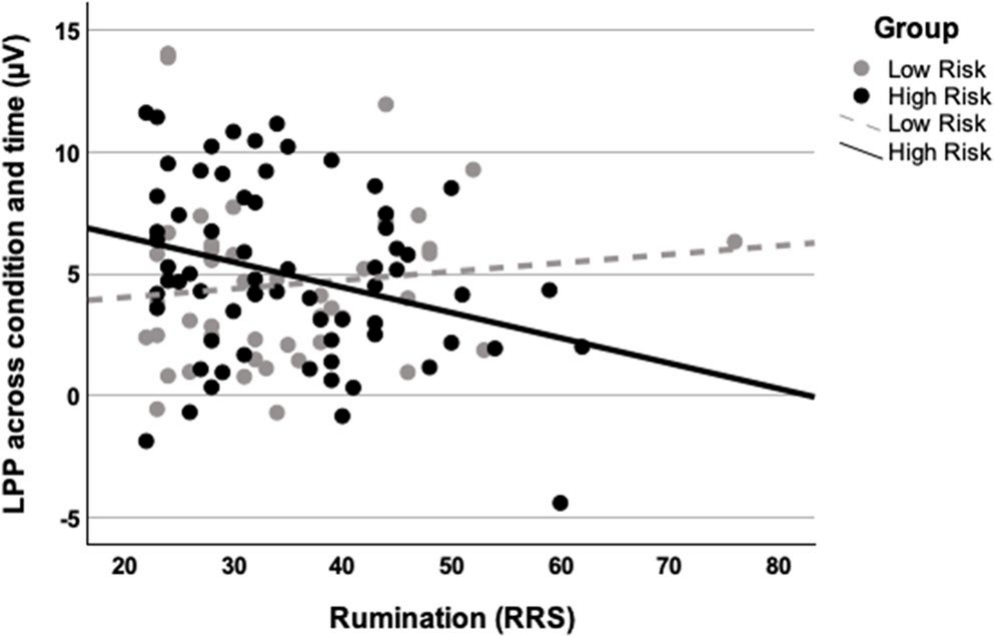
Relation between average late positive potential (LPP) response to emotional faces and shapes (average of angry, fearful, happy, and sad faces and shapes) and ruminative response scale (RRS) among low and high risk youth*.* The main effect of RRS on LPP was significant in the HR youth, *F* (1, 63) = 6.13, *p* =.02, *η*_*p*_^2^ = 0.09, but not among the LR youth, *F* (1, 41) = 0.56, *p* =.46, *η*_*p*_^2^ = 0.01.

Mixed ANOVAs were conducted to test our main hypotheses. As shown in Table 3, when including rumination as a covariate of interest, results revealed main effects of time and age. The LPP response collapsed across conditions and time was negatively correlated with age, *r* = −.35, *p* <.001. Results also revealed a main effect of group, which was qualified by a group × RRS interaction. Further post-hoc analysis revealed that the main effect of RRS on LPP was significant in the HR youth *F* (1, 63) = 6.13, *p* =.02, *η*_*p*_^2^ = 0.09, but not among the LR youth *F* (1, 41) = 0.56, *p* =.46, *η*_*p*_^2^ = 0.01.^1^ Specifically, in HR youth, rumination was negatively correlated with the LPP response across all conditions and time (Fig. 2), *t* = −2.49, *b* = − 0.10, *SE* = 0.04, *p* =.02, whereas the association between rumination and LPP was non-significant in the LR youth, *t* = 0.75, *b* = 0.04, *SE* = 0.05, *p* =.46. This finding was maintained when adjusting for the influence of children’s current depressive symptoms (*F* (1, 63) = 4.49, *p* =.04, *η*_*p*_^2^ = 0.07). Finally, when examining the effects of cognitive reappraisal and suppression in the models, results revealed no significant main effects or interactions with these variables (see Table 3).

**Table 3:** Generalized linear model repeated measures ANOVA results

Rumination Model	F	η_*p*_^2^
Emotion	1.45	0.014
Time	51.39***	0.327
Age	10.78**	0.092
Gender	0.32	0.003
Group	4.42*	0.040
RRS	0.01	0.000
Emotion × Group	0.32	0.003
Emotion × RRS	0.39	0.004
Emotion × Time	0.22	0.002
Time × Group	0.01	0.000
Time × RRS	0.38	0.004
RRS × Group	4.95*	0.045
Emotion × Time × Group	1.68	0.016
Emotion × Time × RRS	1.42	0.013
Emotion × Group × RRS	0.38	0.004
Time × Group × RRS	0.00	0.000
Emotion × Time × Group × RRS	1.79	0.017
**Cognitive Reappraisal Model**	***F***	***η***_***p***_^**2**^
Emotion	2.10	0.019
Time	40.36***	0.276
Age	10.81**	0.093
Gender	0.76	0.007
Group	1.81	0.018
ERQ-Reappraisal	0.20	0.002
Emotion × Group	0.73	0.007
Emotion × ERQ-Reappraisal	1.73	0.016
Emotion × Time	0.53	0.005
Time × Group	0.11	0.001
Time × ERQ-Reappraisal	0.64	0.006
ERQ-Reappraisal × Group	1.97	0.018
Emotion × Time × Group	0.46	0.004
Emotion × Time × ERQ-Reappraisal	0.07	0.001
Emotion × Group × ERQ-Reappraisal	1.04	0.010
Time × Group × ERQ-Reappraisal	0.08	0.001
Emotion × Time × Group × ERQ-Reappraisal	0.57	0.005
**Suppression Model**	***F***	***η***_***p***_^**2**^
Emotion	1.14	0.011
Time	40.41***	0.276
Age	12.19***	0.103
Gender	0.21	0.002
Group	0.01	0.000
ERQ-Suppression	0.97	0.009
Emotion × Group	0.53	0.005
Emotion × ERQ-Suppression	0.57	0.005
Emotion × Time	0.28	0.003
Time × Group	0.47	0.004
Time × ERQ-Suppression	0.39	0.004
ERQ-Suppression × Group	0.49	0.009
Emotion × Time × Group	0.11	0.001
Emotion × Time × ERQ-Suppression	1.89	0.017
Emotion × Group × ERQ-Suppression	0.72	0.007
Time × Group × ERQ-Suppression	0.67	0.006
Emotion × Time × Group × ERQ-Suppression	0.30	0.003

Group = Maternal history of major depressive disorder. *RRS* = Ruminative Response Scale, *ERQ* = Emotion Regulation Questionnaire. * = *p* <.05; ** = *p* <.01; *** = *p* <.001

## Discussion

The current study examined the interplay of maternal depression and youth’s self-reported ER use in association with LPP responses to emotional stimuli among youth. Findings revealed a significant interaction between maternal history of MDD and youth rumination in predicting youth LPP response to all emotions. Specifically, a negative association between trait rumination and LPP response was observed for HR, but not LR, offspring, such that HR youth who reported engaging in higher levels of rumination exhibited a more blunted LPP response to all emotional faces and shapes. Interestingly, no significant interactions were found between maternal history of MDD and suppression or cognitive reappraisal in predicting youth LPP response. Taken together, these findings suggest that the association between maternal MDD history and attenuated electrocortical responses to stimuli, regardless of valence, is dependent on whether HR offspring report engaging in more frequent rumination.

The finding of an attenuated LPP response in HR offspring is consistent with prior research indicating that HR youth are characterized by a blunted LPP (Kujawa et al., 2012; Seidman et al., 2020; Nelson et al., 2015). However, this effect was moderated by rumination, suggesting that HR youth who engage in frequent habitual rumination may be at the greatest risk for altered LPP responses. Given its relatively late time-course, the LPP is thought to index sustained, elaborative processing of affective stimuli, which are processes that overlap conceptually with the repetitive, prolonged, and evaluative qualities of rumination (MacNamara et al., 2009; Watkins & Nolen-Hoeksema, 2014). Thus, it may be that rumination, as a cognitively elaborative form of emotion regulation, modulates later stages of affective processing reflected in the LPP. While previous research has linked youth rumination to increased subjective emotional reactivity (Borelli et al., 2014; Somers et al., 2020), our findings are consistent with a study by Bylsma and colleagues (2022), which reported that greater daily-life rumination was associated with a blunted LPP to unpleasant images in anxious youth. This attenuated LPP response in HR youth may reflect disengagement from emotionally salient stimuli (i.e., faces), a pattern often associated with depressive symptomatology (Weinberg et al., 2016; Klawohn et al., 2021; Foti et al., 2010). Importantly, we found that this attenuated response in youth of depressed mothers was to *all* emotional faces and shapes, rather than valence specific. This generalized attenuation could indicate an early neural marker of risk for MDD among HR youth who engage in frequent rumination. Notably, the lack of valence specificity may reflect a broader vulnerability among this subgroup of HR youth to emotional dysregulation, rather than a focused attentional bias toward specific emotions like sadness, which has been observed in some attention studies of at-risk and currently depressed youth (Gibb et al., 2023; Hankin et al., 2010). Moreover, our findings are consistent with the emotion context-insensitivity hypothesis, which theorizes that MDD may be associated with dampened emotional reactivity to both positive and negative stimuli (Rottenberg et al., 2005).

In contrast to our hypothesis, we did not find significant interactions between the other ER styles, cognitive reappraisal and suppression, and maternal history of MDD in predicting youth LPP response. Given the low internal consistency of the ERQ-CA suppression subscale in this sample, null findings involving this measure should be interpreted with caution, as measurement unreliability may have obscured true associations. Moreover, previous studies linking habitual cognitive reappraisal to reduced LPP responses have primarily been with adult participants (Harrison & Chassy, 2019; Moser et al., 2014), whereas the current study included youth at high and low risk for MDD. Cognitive reappraisal might have differential effects across developmental stages; indeed, there is research showing that adolescents, who are still developing ER capacities, may engage in cognitive reappraisal less effectively or frequently than adults (Garnefski et al., 2002). Our null findings regarding the LPP and cognitive reappraisal are in accordance with previous studies that also found no association between habitual reappraisal and the LPP in anxious youth (Bylsma et al., 2022) and in adults with and without social anxiety disorder (Kinney et al., 2019).

Compared to habitual (e.g., trait-like) ER strategies, it is possible that state reappraisal or suppression induced in the laboratory while the EEG is being recorded may be more significantly associated with the LPP (e.g., Cauwenberge et al., 2017; Dennis & Hajcak, 2009). Specifically, in previous ERP studies assessing the impact of ER strategies on the LPP, authors instructed participants to engage in cognitive reappraisal or suppression while the LPP was being recorded (Li et al., 2020; Yan et al., 2022). In these studies, the LPP was shown to be modified while engaging in these ER strategies during the task across youth and adult participants (Dennis & Hajcak, 2009; Kudinova et al., 2016; Li et al., 2020; Yan et al., 2022). Thus, future work is needed to evaluate how the induction of ER strategies in the laboratory may impact LPP associations in offspring of depressed and non-depressed mothers.

The current study was the first to examine how a variety of self-reported ER styles may interact with maternal MDD history to predict LPP response in offspring. There were some notable limitations which reflect important avenues for future research. First, given the cross-sectional design, future studies would benefit from examining these associations at multiple timepoints to evaluate whether an attenuated LPP prospectively predicts depression symptoms in HR youth and how ER styles may impact this association over time. Second, the current sample was predominantly female (78%), limiting the generalizability of the findings to males. Given evidence that females report more frequent use of rumination, relative to males (Johnson & Whisman, 2013), future studies should have a more balanced sex ratio to examine potential sex differences in the interplay of maternal MDD and rumination in predicting LPP patterns. Third, the timing and duration of mothers’ depressive episodes during the child’s lifetime were not assessed in the current study. Moreover, some of the mothers with MDD also exhibited other forms of psychopathology (e.g., anxiety disorders), which may have impacted study findings. Thus, future adequately powered studies are needed to evaluate how characteristics of maternal depression (e.g., timing, severity, exposure length) and comorbid psychiatric diagnoses may impact offspring’s LPP patterns. Finally, the current study only utilized a self-report measure to assess youths’ ER styles, which may be subjected to bias. Future research should consider instructing participants to employ specific ER styles during EEG tasks while measuring the LPP, as done in prior studies (e.g., Hajcak & Nieuwenhuis, 2006). Additionally, future research should consider employing methods that assess ER styles in real time, such as ecological momentary assessment, to provide a more accurate and dynamic understanding of youths’ ER styles in their daily lives (Ebner-Priemer & Trull, 2009).

In summary, the present study contributes to the LPP literature by examining how various ER styles interact with maternal history of MDD to predict LPP response in youth. Specifically, we found that HR youth who reported higher levels of rumination displayed an attenuated LPP in response to all emotional faces, suggesting that engaging in rumination may heighten vulnerability to altered emotion processing in HR youth. Findings suggest that prevention programs aimed at altering emotion processing skills might be most beneficial for HR youth who habitually ruminate.

## Data Availability

The data that support the findings of this study are available from the corresponding author upon reasonable request.

## Footnotes

1 We also examined rumination as the moderator in simple slope analysis. The association between group and LPP response across all conditions and time was significant for youth reporting higher (+ 1SD, *t*=-2.56, *p* <.05, *b*=-1.78), but not lower (-1SD, *t*=-1.45, *p* =.10, *b* = 1.45), rumination.
